# SARS-CoV-2: Pathogenesis, and Advancements in Diagnostics and Treatment

**DOI:** 10.3389/fimmu.2020.570927

**Published:** 2020-10-06

**Authors:** Khalil Khalaf, Natalia Papp, Jadzia Tin-Tsen Chou, Doris Hana, Andrzej Mackiewicz, Mariusz Kaczmarek

**Affiliations:** ^1^Department of Cancer Immunology, Poznan University of Medical Sciences, Poznań, Poland; ^2^Department of Cancer Diagnostics and Immunology, Greater Poland Cancer Center, Poznań, Poland

**Keywords:** SARS-CoV-2, COVID-19, spike receptor, ACE2, non-structural protein, remdesivir, EMMPRIN, monoclonal antibodies

## Abstract

The emergence and rapid spread of SARS-CoV-2 in December 2019 has brought the world to a standstill. While less pathogenic than the 2002–2003 SARS-CoV, this novel *betacoronavirus* presents a global threat due to its high transmission rate, ability to invade multiple tissues, and ability to trigger immunological hyperactivation. The identification of the animal reservoir and intermediate host were important steps toward slowing the spread of disease, and its genetic similarity to SARS-CoV has helped to determine pathogenesis and direct treatment strategies. The exponential increase in cases has necessitated fast and reliable testing procedures. Although RT-PCR remains the gold standard, it is a time-consuming procedure, paving the way for newer techniques such as serologic tests and enzyme immunoassays. Various clinical trials using broad antiviral agents in addition to novel medications have produced controversial results; however, the advancement of immunotherapy, particularly monoclonal antibodies and immune modulators is showing great promise in clinical trials. Non-orthodox medications such as anti-malarials have been tested in multiple institutions but definitive conclusions are yet to be made. Adjuvant therapies have also proven to be effective in decreasing mortality in the disease course. While no formal guidelines have been established, the multitude of ongoing clinical trials as a result of unprecedented access to research data brings us closer to halting the SARS-CoV-2 pandemic.

## Introduction

Coronaviruses are widely known virulent pathogens affecting mammalian and avian species. Previously, six globally distributed species of the virus have been identified to cause illness in humans. They are: human coronavirus OC43 (HCoV-OC43), human coronavirus HKU1 (HCoV-HKU1), Human coronavirus 229E (HCoV-229E), human coronavirus NL63 (HCoV-NL63), Severe acute respiratory syndrome coronavirus (SARS-CoV), and Middle East respiratory syndrome coronavirus (MERS-CoV) (Table 1).

**Table 1:** Pathogenic coronaviruses in recent history.

**Alphacoronaviruses and related**		
Human coronavirus 229E	HCoV-229E (1, 2)	Mid-1960's−5–30% of upper respiratory tract infections
Human coronavirus NL63	HCoV-NL63 (3)	Early 2000's—community-acquired pneumonia of the lower respiratory tract
**Betacoronaviruses**		
Human coronavirus OC43	HCoV-OC43 (1, 2)	Mid-1960's−5–30% of upper respiratory tract infections
Human coronavirus HKU1	HCoV-HKU1 (3)	Early 2000's—community-acquired pneumonia of the lower respiratory tract
Severe acute respiratory syndrome coronavirus	SARS-CoV (4)	2002–2003, China
Middle East respiratory syndrome coronavirus	MERS-CoV (4)	2013, Saudi Arabia
COVID-19	SARS-CoV-2	2019, Wuhan, China

### SARS-CoV

SARS-CoV first emerged in 2002–2003 in China, presenting as an atypical pneumonia with febrile state, headaches, and a marked cough that may rapidly deteriorate into respiratory failure. The SARS-CoV outbreak was the first to be declared a pandemic, and ultimately infected 8,000 persons in 29 countries, with a mortality rate of 10% (5). Epidemiological analysis posited an infectious zoonotic agent, and further serological studies identified the intermediate host to be the palm civet (*Paguma larvata*). Spillover from animals to humans was hypothesized to be via the horseshoe bat (genus *Rhinolophus*). An adaptation of the SARS-CoV virus was later proven to be due to interspecies dwelling (6).

A combination of ribavirin and pulse prednisolone showed positive results early in the outbreak and was adopted as the standard protocol. Eventually, ribavirin was demonstrated to have increased cytotoxicity and lacked efficient antiviral action *in vitro*, and while pulse prednisolone showed efficacy in managing critically ill patients, its high dose administration was associated with disseminated fungal infections (7). Later, the combination of interferon-alpha1 (INF-α1) and corticosteroids was found to yield a better prognosis, but did not prove beneficial for patients in the late stage of the disease (8). Protease inhibitors produced an outcome similar to that of INF-α1 (9).

### MERS-CoV

A novel coronavirus later named MERS-CoV emerged in Saudi Arabia in 2012. Similar to SARS, infected patients presented with a variety of clinical courses, from mild upper respiratory symptoms to fulminant pneumonia and multi-organ system failure. Phylogenic and sequencing studies have proposed a bat origin, and surface protein modification was found to be derived from the intermediate hosts, dromedary camels. From 2012 to 2015, more than 2,000 confirmed cases were reported, mainly in Middle Eastern countries, with a mortality rate of 35% (10). As with SARS-CoV, no definitive protocol was implemented, and most patients were treated with supportive therapy to preserve organ integrity (11). In a cohort study conducted by Omrani et al., a treatment protocol involving IFN-α2 and ribavirin was initiated, and while survival improved significantly at 14 days after treatment, this was not seen at day 28, necessitating further treatment options (12). Sporadic outbreaks occur to this day, with patients undergoing largely supportive therapy.

### SARS-CoV-2

The SARS-CoV-2 outbreak began in December 2019 in Wuhan, China where clusters of atypical pneumonia yielded evidence of a novel strain of coronavirus. As of August 24, 2020, 16,036,308 cases had been reported worldwide, with a fatality rate of 5%. Although this novel virus is less severe than the first SARS-CoV outbreak, human-to-human transmission remains very high and the number of cases continues to rise exponentially in major urban areas, highlighting the urgent need to develop new containment, diagnostic, and treatment protocols. As time is of the essence, the pathogenic mechanism cannot be studied with the rigor and comprehension otherwise expected, and the opportunities to compare therapeutic protocols are few.

## SARS-CoV-2

### Origin of the Virus

During the initial outbreak, Liu et al. obtained samples of blood, bronchoalveolar lavage fluid, and anal and oral swabs from patients in Wuhan, China suffering from a pneumonia-like illness resembling the 2002–2003 SARS-CoV. A pan-CoV PCR was performed on samples from five patients, and were positive for a pathogen from the *Coronaviridae* family. This was compared with a full-length sequence of viral RNA from a bat coronavirus (bat-CoVRaTG13), and demonstrated 96.2% similarity. Thus, it is probable that the bat is the main reservoir of the novel coronavirus. Identification of the intermediate host is an essential step in controlling the spread of disease, and became a priority for research teams. Unfortunately, this was complicated by the many species of wild animals sold at the Huanan seafood market, where the first cases were reported to have had contact. In 2019, a SARS-CoV-like pathogen known to be widely distributed in the Malayan pangolin samples was discovered. The receptor-binding domain (RBD) present on the spike protein (S) is a crucial determinant in host range, as its interaction with the host receptor is responsible for the infection. RBD sequences from bat-CoVRaTG13, pangolin-SARS-like CoV and the novel SARS-like pathogen were aligned. Ninety three percentage similarity was demonstrated between the novel SARS-like pathogen and the pangolin SARS-like CoV, and 89% similarity was demonstrated between the novel SARS-like pathogen and the bat-CoVRaTG13. Thus, on the basis of the RBD, the pangolin-SARS-like CoV is determined to be more likely than the bat-CoVRaTG13 to infect humans, making this the possible intermediate host (13). Xiao et al. conducted another study in which the pangolin-SARS-like CoV was isolated and amino acid sequence was compared to SARS-CoV-2. This yielded 100, 98.6, 97.8, and 90.7% similarity with the S, M, E, and N proteins, respectively, of the novel SARS-CoV, strengthening the previous assumption that the pangolin was the intermediate host (14).

### Official Classification of the Virus

Pathogenic classification is used to determine whether the pathogen is new or recurring in order to best implement safety and treatment protocols. While serological reactivity to viral proteins had been the mainstay of viral classification in the past, the process today now depends on replicated protein sequences. The International Committee on Taxonomy of Viruses (ICTV) maintains a study group for each viral family (15). After analysis, the novel virus was assigned to the order *Nidovirales* on the basis of the following domains: polyprotein protease (3CLpro), catalytic domain of RNA polymerase (RdRp), Nidovirus-associated RdRp (NiRAN), zinc binding domain (ZBD), and helicase (HEL1) (16). Subsequent next generation sequencing and phylogenic analysis placed the novel pathogen within the subgenus *Sarbecovirus* of the genus *betacoronavirus* (17) (Table 2).

**Table 2:** Classification of SARS-CoV-2 (18).

**Systematics**	
Order	*Nidovirales*
Family	*Coronaviridae*
Genus	*Betacoronavirus*
Subgenus	*Sarbecovirus*
Species	SARS-related coronavirus
Naming authority	SARS-CoV-2

### Virion Structure

Like other coronaviruses, the viral envelope is composed of a lipid bilayer derived from host cellular material, and the structural proteins are spike proteins composed of a trimeric glycoprotein projecting from the envelope like a crown. Cryo-electron microscopy was used to compare the structural differences between the spike (S) proteins of SARS-CoV and SARS-CoV-2. Unlike the previous SARS-CoV which possessed a single arginine targeted by a trypsin protease, SARS-CoV-2 possesses an S1/S2 protease cleavage site which is recognized by furin proteases. Neuman et al. demonstrated the high level of protein organization and interaction within the envelope: the S protein was shown to be aligned with the NPC, a crucial component of viral organization in protein-protein interactions that ensures high specificity and effectiveness for host invasion (19).

### Viral Genome and Replication

SARS-CoV-2 is an enveloped virus with a 29,904 bp positive-sense non-segmented RNA genome. The non-structural proteins (NSPs) are described in Table 3. The remainder of the genome is responsible for accessory and structural proteins such as S, M, E and N.

**Table 3:** Non-structural proteins of SARS-CoV-2 and their functions (20).

**Non-structural proteins**	**Functions**
Nsp1	Host mRNA degradation, inhibit IFN signal
Nsp2	N/A
Nsp3	Polyprotein processing via papain-like protease, Structure TM domain, inhibits ubiquitination; DMV?
Nsp4	DMV formation? TM domain
Nsp5	Polyprotein processing via 3C-like proteinase
Nsp6	DMV formation? TM domain
Nsp7	Single-stranded RNA binding
Nsp8	Primase
Nsp9	Component of replicase complex
Nsp10	Component of replicase complex
Nsp11	N/A
Nsp12	RNA dependent RNA polymerase
Nsp13	Helicase action, ZBD
Nsp14	Exoribonuclease activity, 5'-cap formation (N-7-methyltransferase)
Nsp15	Endoribonuclease activity
Nsp16	5'-cap formation (2-O-methyltransferase)

### Replication

Host cell recognition is the first and most essential step in viral pathogenesis. Studies on the 2002–2003 SARS-CoV outbreak uncovered key interactions between the spike (S) protein, the RBD and angiotensin-converting enzyme 2 (ACE-2). Due to the previously mentioned similarities between SARS-CoV-2 and its predecessor, a viral infectivity study was performed using HeLa cells with and without the ACE-2 receptor, which showed that only cells possessing the ACE-2 receptor were infected (21). The spike trimer is a class I fusion protein; upon infection the spike is cleaved by host proteases at the S1/S2 site for division of the two domains. The S1 domain possesses the RBD which recognizes and binds the ACE-2 receptor in a prefusion state. Structural rearrangement of the S protein subsequently exposes a furin cleavage site on the S2 domain which enables viral entry by means of fusion after the S1 domain is shed (22). Viral replication was hypothesized to occur via a process called autophagy: an evolutionary cellular process in which cytoplasmic proteins create isolation membranes surrounding materials destined for degradation (23). Evidence of coronavirus autophagy was first demonstrated by Prentice et al., who showed that in coronavirus mouse hepatitis viruses, ATG5-induced autophagy was required for the formation of double membrane vesicles (DMV) and for replication (23). Another study confirmed the use of ATG5-dependent autophagy of betacoronaviruses through NSP 6 (24). Chen et al. previously showed that both SARS-CoV and MERS-CoV employ the use of Papain-like Proteases (PLpro) to induce the formation of autophagosomes, but their fusion to lysosomes is inhibited which promotes the replication process (25).

However, other studies have demonstrated that key autophagy proteins ATG5 or ATG7 were not required for coronavirus mouse hepatitis or SARS-CoV viral replication (26). Reggiori et al. also yielded comparable results showing that the replication and release processes are not dependent on autophagy, but that the DMV's were coated with (LC3)-I (non-lipidated microtubule associated protein I light chain 3), thus showing the importance of this protein for viral replication (27).

A recent study by Benveunto et al. has shown that SARS-CoV-2 induced mutations within the NSP6 protein, which in turn induced autophagosome formation by inhibiting phagosome-lysosome fusion (28, 29). SARS-CoV-2 may take advantage of this autophagy mechanism, as the acidic pH required for endosome maturation also functions to release the virion at the appropriate site. Once all necessary proteins are translated and replication has taken place, virion assembly occurs in the Golgi apparatus before release from the cell (30).

## Clinical Presentation

### Symptoms Attributed to SARS-CoV-2

Shereen et al. determined a mean incubation period of 5 days in the first 425 patients testing positive for SARS-CoV-2 (31). The time from infection to death varied from 6 to 41 days, with a mean of 14 days (32). In January of 2020, Huang et al. examined all patients exhibiting pneumonia-like symptoms in order to determine the clinical features of SARS-CoV-2; importantly, this study did not discriminate between previously healthy patients and patients suffering from chronic illnesses. Data was collected from the 41 positive cases, and analysis yielded the following: fever was the first and most common primary symptom (98%), followed by dry cough (76%), lymphopenia (63%), dyspnea (55%), and fatigue/myalgia (44%). Less common symptoms included sputum production (28%), headache (8%), hemoptysis (5%), and diarrhea (1%) (33). A recent systematic review of 148 studies spanning nine countries disputes the above-mentioned data. In this analysis by Grant et al., the most common symptoms were as follows: fever (78%), dry cough (57%), fatigue (31%), anosmia (25%), and difficulty breathing (23%) (34). Another study by Clemency et al. assessed the likelihood that SARS-CoV-2 infection presents with symptoms. The positive likelihood ratio of having symptoms with disease from highest to lowest was as follows: anosmia and ageusia, followed by loss of appetite, fever, muscle pain, fatigue, dry cough, productive cough, diarrhea, difficulty breathing, and sore throat (35).

### Mechanism of Pathogenesis

#### Ease of Transmission

The stability of SARS-CoV-2 on various surfaces was compared to that of its predecessor SARS-CoV, and it was found that both viruses exhibit similar stability. The differences in epidemiological spread are therefore most likely to be dependent upon other factors such as high viral load and viral shedding in asymptomatic persons. The study is highly suggestive of an aerosolized pathogen as it remains viable and infectious in droplet form for up to 3 h (36). While resembling the previous SARS-CoV outbreak, this data confirms that SARS-CoV-2 is far more transmissible. Kinetic studies employed to compare the two strains showed that SARS-CoV-2 binds to the ACE-2 receptor with a 10- to 20-fold higher affinity than SARS-CoV, suggesting that this could be another main factor explaining the ease of transmission (37). To further support this hypothesis, computational structural predictions were established to differentiate between the rate of association between the ACE-2 receptor and the spike protein of both SARS-CoV and SARS-CoV-2. By incorporating the computer simulation into a mathematical model, it was shown that the novel pathogen has slower binding capacity than SARS-CoV, consistent with the varying life cycles of the two strains. In other words, the longer incubation period of SARS-CoV-2 was associated with the slower interaction between the spike protein and the ACE-2 receptor. This allows for maintenance of an increased viral load in the body, thus contributing to increased human-to-human transmission (38).

#### Tissue Tropism

The renin-angiotensin-aldosterone system (RAAS) is responsible for inducing a coordinated cascade regulating both cardiovascular and renal functions. Angiotensin-converting enzyme (ACE) is responsible for converting angiotensin I to angiotensin II, with the latter functioning as a pro-sympathetic molecule throughout the body to regulate blood pressure. Wakahara et al. measured ACE and ACE-2 expression under various conditions. In addition to the ACE-2 role in local regulation of RAAS through the conversion of angiotensin II into vasodilators and anti-trophic peptide, it acts and is expressed in synergy with ACE concentrations and is believed to possess counter-regulatory effects to ACE (39). As the expression of the ACE-2 receptor is vital for viral entry, it is essential to establish where this gene is expressed throughout the body to identify possible routes of infection and/or the extent of organ damage during infection. Zou et al. employed the dataset systems of single-cell RNA sequences from different body tissues to identify organs expressing the ACE-2 gene in high concentrations. The symptoms reported to be associated with COVID-19 (dyspnea, cardiac injury, kidney failure, diarrhea) were compared to ACE-2 expression on target cells. It is on this basis that the organs at high risk of damage during viremia were recognized to be the lungs, heart, kidney, and upper respiratory tract (40). A minority of patients is seen to present with gastrointestinal involvement, and a further study by Wong et al. on 140 patients in Wuhan demonstrated this complaint in 39% of their cohort. The virus was detected in stool by means of RT-PCR. Staining of the N protein showed presence of the virus in the cytoplasm of duodenal, rectal and gastric epithelium thus indicating another possible mode of transmission and another sign of possible infection (41). Zhao et al. also compared the symptoms and disease characteristics of COVID-19 patients with that of other pneumonias, indicating that liver damage was more prominent in COVID-19 patients. However, it was not clear whether liver enzyme elevation was due to drug toxicity or viral shedding (42).

The ACE-2-receptor mapping experiment, while important, provides little information on the broad expressions of the receptor, as ACE-2 concentration may differ due to various clinico-pathologies such as hypertension, diabetes, heart failure, smoking and kidney injury (43, 44). Remuzzi and Remuzzi in Italy demonstrated that two-thirds of patients who died from SARS-CoV-2 were elderly, diabetic or suffered from cardiovascular disease (45). Their first line drug of choice was angiotensin receptor blockers (ARBs). Ferrario et al. demonstrated in 2005 that selective blockade of angiotensin II synthesis or activity has an up-regulatory effect of ACE-2 receptors in the kidneys and heart (46). This ACE-2 upregulation in the aforementioned comorbidities could therefore be responsible for the severity of infection in these patients. Guo et al. demonstrated that in 187 SARS-CoV-2-positive patients, 28.7% had an increase in troponin-T and CRP levels that correlated with myocardial injury. SARS-CoV-2 patients who presented with an increase in cardiac markers exhibited a 59.6% mortality rate compared to those with normal cardiac markers (8.9% mortality rate). SARS-CoV-2 genomic material has also been isolated from cardiac biopsies. While the pathophysiologic development of myocardial injury is not yet fully understood, it is believed to be caused by either cytokine storm or direct myocardial damage through viral integration (47).

To further understand the organ damage and hemostatic changes, Han et al. assessed the change in coagulation proteins in both COVID-19 patients and healthy patients. They found that antithrombin levels were lower in COVID-19 patients, whereas fibrinogen, fibrinogen/fibrin degradation products and d-dimer were high (48). A recent cohort study by Wichmann et al. reported a possible connection between COVID-19 and incidence of thromboembolism. Autopsies of 12 COVID-19 patients revealed the incidence of deep vein thrombosis (DVT) in 58% of cases, and a third of patients suffered a fatal pulmonary embolism. Further studies should be conducted to further elucidate the formation of thromboembolism in the course of COVID-19 (49).

CD147, also known as EMMPRIN (Extracellular matrix metalloproteinase inducer) is a highly glycosylated transmembrane glycoprotein shown to be involved in facilitating SARS-CoV endocytosis. This presents an alternative route of viral invasion (50). Like the ACE-receptor, CD147 is also present in various tissues such as lymphoid (51), testes, liver, cerebral, and renal tissues, as well as cardiac and skeletal muscle (52). In addition to enabling viral entry, CD147 has been linked to other pathologic conditions such as cancer (53, 54), heart disease (55) and Alzheimer's disease (56). Furthermore, the speed of invasion may be increased as CD147 has been shown to be upregulated upon T-cell activation (57). Wang et al. has since shown that the spike protein possesses high affinity for CD147, thus mediating viral invasion and facilitating viral replication. It acts as a negative regulator of the immune system which may contribute to the significant CD4+ decline during COVID-19 (58). Under hypoxic conditions, this receptor is involved in the regulation of cell proliferation and apoptosis. The CD147 receptor, a marker of early-stage disease, can be useful for the assessment of pathological progression because it is often expressed on the surface of activated regulatory T cells (59).

#### Immune Evasion

##### Innate immunity

Lung epithelium is the largest area of the body in constant contact with the external environment, and is responsible for processing inhaled air containing large volumes of bacteria and viruses. The immune response to SARS-CoV was widely studied, and SARS- CoV-2 is believed to induce the same responses. Innate immunity is the primary countermeasure against infection, and is maintained by a variety of cell types found in airway epithelium, including dendritic cells, innate lymphoid cells and alveolar macrophages. The protective signaling cascade begins as the pattern recognition receptors (PRRs) of innate cells recognize pathogen-associated molecular patterns (PAMPs) of viruses. After recognition, type I and III interferon (IFN) and other pro-inflammatory cytokines undergo transcription. Autocrine and paracrine signals in both healthy and infected cells subsequently ensure the translation of these cytokines (60).

##### Passive evasion

Passive evasion refers to any mechanism of immune avoidance that does not directly interfere with the host immune system. Many non-structural proteins (NSP) are translated in order to shield viral genomic material from the human immune system, as shown in Table 3. NSP 3, 4, and 6 are believed to function as modifiers of the intracellular membranes of organelles, where the virus may safely replicate. Another passive mechanism is the conformational change of the viral mRNA through either the addition of a 5'-cap (via formation of N-7-methyltransferase in NSP 14), or a 2'-O-methyl-transferase as with NSP 16, which marks the viral mRNA to be of host origin. In another example, NSP 15 induces endoribonuclease activity against viral mRNA at certain stages of its development to avoid detection (20).

##### Active evasion

NSP 1 was shown to inhibit host mRNA translation through the activation of exonucleases. These induce degradation of the host genome and prevent the IFN signal transduction that is necessary to activate viral clearance. NSP 3 expresses PLpro, which greatly resembles host cellular de-ubiquitinase action. This induces disruption of ubiquitin-mediated degradation and may also inhibit the innate immune response (61).

##### Adaptive immunity

As with the previous outbreak, immune clearance of SARS-CoV-2 relies mostly on the activity of Th1 cells via the extensive secretion of IL-2, IL-10, IFN-γ, and TNF-α/β. This cytokine microenvironment activates macrophages and causes cytotoxic T-cell proliferation, initiating pathogen clearance. Following degradation and the presentation of viral antigens on APCs, the humoral response acts to limit future infections through antibody neutralization (62).

##### Evasion of adaptive immunity

In the lungs, alveolar macrophages are the first cells to make contact with microorganisms. Their main function is to destroy any invaders without overstimulating the adaptive immune system, since foreign pathogens are ever-present. Protective measures employed by alveolar macrophages include the surface expression of CD200 and TGF-β receptors, which function as negative regulators of dendritic cells and T cells (63, 64). This inactivation is exploited by SARS-CoV, leading to an increase in viral load.

Additionally, Law et al. demonstrated that macrophages and dendritic cells are targeted by SARS-CoV, resulting in a very low expression of antiviral cytokines. This inhibits apoptosis and hinders the bridging of the innate and adaptive immune systems, leading to lymphopenia (65). In a cohort study by Qin et al. of patients with confirmed SARS-CoV-2 infection, lymphopenia and an increase in neutrophil-to-lymphocyte ratio was routinely observed among infected patients and was especially evident in severe cases (66). It has been shown that these two parameters are indicative of systemic inflammation, and were associated with the worst prognosis (67, 68). Because Qin and colleagues did not observe any changes in either CD8+ or B cells, it was then hypothesized that SARS-CoV-2 plays a major role in indirectly damaging lymphocytes, with the release of pro-inflammatory cytokines and chemokines that results in the consumption of the lymphocytes necessary to prevent innate overactivation (69). Qin et al. also noted that the increase in neutrophil-to-lymphocyte ratio was accompanied by an increase in procalcitonin, indicating bacterial co-infection (66). Zhao et al. showed that mice depleted of alveolar macrophages and subsequently infected with a mouse-adapted SARS-CoV strain quickly developed activation of dendritic cells, which in turn activated cytotoxic T cells and initiated viral clearance (70).

While most viral infections end with eradication and development of immunological memory, adaptive immunity does on occasion fail to develop adequately. Callow et al. demonstrated in 1990 that patients previously infected with Human coronavirus 229E showed a decline in antibody concentration and were capable of being re-infected after 1 year (71). Other studies involving MERS-CoV have come to the same conclusion, determining that the concentration of virus-neutralizing antibody was dependent upon disease severity (72, 73). It is important to note that the failure of adaptive immunity could be due to either insufficient antibody response or decrease in T-cell durability (74, 75). Appropriate adaptive immunity requires early CD8+ and CD4+ responses. In the case of SARS-CoV-2, viral evasion of the innate immune system leads to an increase in cytokine production and late CD4+/CD8+ response, which then leads to pathogenic inflammation in patients with high viral loads.

#### Disruption of Immune Homeostasis

Due to rapid and unopposed SARS-CoV-2 replication, CD4+ T lymphocytes are quickly activated to differentiate into Th1 cells and are responsible for releasing pro-inflammatory cytokines IL-6, GM-CSF, and IFN-γ. GM-CSF activates to further produce inflammatory monocytes (CD14+ and CD16+) which release more IL-6. This disrupts the homeostasis of the immune system leading to cytokine storm (76). SARS-CoV-2-related hyper-inflammation involves very high levels of IL-1-β, IL-6, and TNF-α (77).

SARS-CoV-2 is thought to bind to the toll-like receptor (TLR), activating inflammasomes and resulting in the cleavage of pro-IL-1- β to form IL-1- β, a mediator of inflammation, fever and lung injury (78).

The pathological immune response has a wide variety of clinical presentations, from mild symptoms to pulmonary failure and death. Extensive lung damage is associated with neutrophil and macrophage infiltration while CD4+ and CD8+ count in peripheral blood are simultaneously lowered (79). Croft et al. performed serological analyses in both healthy and SARS-CoV-2-positive individuals. The CD4+ T cells in infected individuals demonstrated very high levels of CD69, CD44, and CD38, indicating a hyperactive state compared to the healthy group. Further analysis showed high levels of the T-cell activation marker OX40 on CD4+ cells, which has been proven to be crucial for cell expansion, survival and cytokine production in both T cells and innate cells (80). It is important to note that expression levels of OX40 also varied depending on the severity and progression of the disease, and may possibly be a marker of poorer prognosis. CD8+ T cells were also found in a hyperactive state, with higher expressions of CD69, CD44, and CD38.

In various deteriorating ICU patients, there was an increase in expressions of PD-1 and Tim-3 in both CD4+ and CD8+ T cells, indicating immune exhaustion (76, 81). Aside from exhaustion antigens such as PD-1, Bellesi et al. (82) revealed upregulated expression of the CD95 antigen on both CD4+ and CD8+ T cells. When CD95 apoptosis-related antigen is observed to be highly expressed, this is accompanied by a lower CD4+ and CD8+ count, which may partially explain the loss of lymphocytes in SARS-CoV-2-positive patients. The loss of naive cells appears to be particularly important in this context.

Complement activation can be initiated via the alternative pathway, classical pathway or lectin pathway. Innate immunity recognizes pathogen-associated molecular patterns (PAMPs) in host cells and initiates a destructive response. The mannose-binding lectin (MBL) pathway has been shown to be the first line of defense against SARS-CoV (83). It is composed of pattern recognition molecules associated with serine protease from the MBL-associated serine proteases (MASPs), which circulate as zymogens and become active after recognition, binding to carbohydrate-based PAMPs (84). To further understand the triggers of the excessive immune response to SARS-CoV-2, Gao et al. showed that as with SARS-CoV, MASP-2 contact with the N protein led to cleavage of C4 and C2 into C4a/C4b and C2a/C2b, respectively, and the MBL pathway proceeded as normal. None of the other pathways triggered complement activation (85).

## Testing Protocols

Due to the high infectivity and severity of the virus, the World Health Organization (WHO) has been prompted to develop new tools to ensure the fast and accurate diagnosis of the viral infection. Many governments have given an unprecedented level of freedom to nominated laboratories to establish reliable diagnostic tests. The increased demand for rapid testing has fueled research in sequencing, characterizing, and understanding SARS-CoV-2, in order to definitively diagnose it in human samples.

As of August 2020, the recommended sampling sites are as follows: the upper respiratory tract (nasopharynx, oropharynx, anterior nares), lower respiratory tract (sputum, bronchoalveolar lavage, pulmonary tissue biopsy) as well as urine, feces or whole blood. According to Centers for Disease Control (CDC) guidelines, material from the upper and lower respiratory tracts are preferred, and should be sampled if possible. Material should be collected by an experienced specialist to ensure high standards, and should be tested as soon as possible; exceptions are serum samples which can be stored for up to 2–4 weeks, to be referenced in future follow-up (86). The overwhelming number of infected cases compounded with the shortage of healthcare personnel has led to the prioritization of testing of affected individuals. Due to limited access and resources, the CDC has been compelled to define specific criteria for testing all 2019-nCoV patients under investigation (PUI). In order to be tested, the PUI needs to manifest with:

– “Fever and symptoms of lower respiratory illness (cough, difficulty breathing) AFTER 14 days of travel to Wuhan City OR contact with a 2019-nCoV PUI within the last 14 days,” or
– “Fever OR symptoms of lower respiratory illness (cough, difficulty breathing) AFTER contact with a patient with a confirmed case of SARS-CoV-2 infection within 14 days” (87).

The introduction of restrictions not only guarantees good quality patient care and prioritizes acute and severe cases, but maintains the integrity of an already strained healthcare system.

The high volume of cases demands fast and efficacious testing regimes for a variety of settings, including the hospital. Depending on the type of technology and the personnel available, a few specialized diagnostic protocols have proven to be useful in the field.

### Molecular Testing

SARS-CoV-2 is an RNA virus which sheds detectable genetic material in almost all excretions of an infected individual. This material can be detected by a simple nucleic acid test which is capable of identification and characterization of nucleotide sequences. In blood, sputum and other samples, the amount of genetic material is very sparse thus necessitating an additional amplification step in order to reach a particular detection threshold. This method is known as the nucleic acid amplification test (NAAT). Another technique currently available is the polymerase chain reaction (PCR). Real-time polymerase chain reaction (rRT-PCR) is the gold-standard molecular technique for detection of SARS-CoV-2 viral RNA in all recommended samples. It is a primary diagnostic test that targets the following sequences that code for structural viral proteins: spike (S), membrane (M), envelope (E), nucleocapsid (N), and RNA-dependent RNA polymerase (RdRP). The available literature suggests that the spike protein (S) may be the primary pivot in intracellular interactions with host cells, thus demonstrating high immunogenic character (88). High infectivity of SARS-CoV-2 has compelled the CDC to publish rRT-PCR primers and probes together with all relevant literature for public access (87). Such advances in research were possible thanks to past experience with the previous *betacoronavius* epidemic. Primer design was based on the nucleotide sequences that matched SARS-CoV and MERS-CoV with 80 to 90% accuracy (21). The wide availability of protocols has accelerated progress in research and diagnostic measures. Nevertheless, it should be noted that the high mutation rate and large genetic variability of the virus may negatively affect the performance of the assay, and may lead to an increasing number of false-negative results (89).

Additionally, the difficulty of the assay, complexity of the logistic analysis, and protocol duration (45 min to a few hours) (90) confer some limitations to this diagnostic tool (62). The full RNA extraction protocol should be implemented in a biosafety cabinet at BSL-2 security level by trained and skilled personnel. It is recommended that none of the samples be heat-treated before RNA extraction, which means that samples pose a high risk of infection to laboratory technicians (86). False-positive results may also be obtained in cases where the amount of viral material in a collected sample is too low for detection (89).

Based on a published summary report by the FDA, the analytical sensitivity of RT-PCR is 80% with a limit of detection of 6.25 cp/uL. Specificity showed no cross-reactivity with the most common pathogens: bacterial (*Legionella pneumophilia, Mycobacterium tuberculosis* and *M. pneumoniae*, and *Streptococcus pneumoniae* and *S. pyogenes*) and viral (adenovirus, parainfluenza, rhinovirus, RSV, etc.). The only detected cross-reactivity corresponded to the Severe Acute Respiratory Syndrome coronavirus (SARS-CoV), but this is not surprising since the N3 target gene in SARS-CoV-2 demonstrates more than 80% genetic similarity to other *betacoronaviruses* (91).

The diagnostic protocol formulated by the CDC clearly states the process of confirmation of SARS-CoV-2 infection, though the criteria depend on the area where the PUI is being diagnosed. In areas where the concentration of SARS-CoV-2 virus is high, a single positive NAAT result is required to diagnose the patient. In areas with low viral circulation more than one of the following is required:

– positive NAAT for at least two different targets on the SARS-CoV-2 genome (one specific for the virus)
– 1 positive NAAT for *betacoronavirus* (SARS-CoV, MERS-CoV, SARS-CoV2) with SARS-CoV-2 genome sequencing. It is indicated that the sequenced fragment must be longer or different from the fragment used in the NAAT assay (86).

In cases where all tested controls are positive, with all SARS-CoV-2 markers below growth thresholds, the NAAT result is negative. However, cases with high clinical suspicion may still produce a negative RT-PCR result. This may be due to a number of factors, such as poor quality and handling of specimens, specimen collection too early or late in the disease course, contamination, and/or technical errors (86). In other words, the lack of a positive result does not exclude COVID-19 disease. In such instances identification of the infected individual is based on clinical observation, patient history and epidemiological information. Given the number of tests done to date, in the case of discordant opinions, re-sampling and use of different markers is advised (86).

### Immunoassays

The limitations of NAAT diagnostic techniques for SARS-CoV-2 have generated increasing demand for quicker and simpler tests based on serology. RT-PCR, while very reliable and precise, is only capable of identifying the presence of viral load, and cannot inform on the state or progress of the infection in a given patient. In contrast, serological testing and enzyme-based testing measure immunological responses to the virus, allowing for differentiation between exposed asymptomatic, acutely or mildly sick, and recovered cases. Additionally, it is able to quantify the number of cases within a short period of time, and this is crucial for modeling the population-scale of infection, determining the level of prophylaxis, and has implications for the development of a vaccine against SARS-CoV-2.

There are many immunoassay techniques currently approved or pending. Their versatility and creativity augment the number of ways with which one can detect the pathogen in a studied sample. They can be divided into serologic tests and enzyme-based immunoassays. Serologic tests exploit the natural responses of the human immune system, while enzyme-based immunoassays detect the antigen with specifically manufactured monoclonal antibodies.

#### Serologic Tests

All tests in this category detect either the antigen shed by the virus or the antibody that is produced by B-cells in response to the pathogen. The rapid diagnostic test (RDT) is a serologic type of diagnostic tool that allows for detection of the target after 10–30 min (90). It is a qualitative lateral flow type assay that resembles the standard rapid chromatographic test used for the detection of beta-hCG hormone in the urine of pregnant women. As a field-based test, it requires a drop of blood or other body fluid for identification of IgG and/or IgM antibodies, or the viral antigen itself (90). Antigen based-RDTs target the structural viral proteins (S, N, etc.) mainly found in secretions of the upper respiratory tract, and react to the antibodies that are produced 10–30 days after the initial infection. Sufficient time is required for the concentration of antibodies to reach the threshold of detection (92). It is also advised to measure IgG and IgM titer baselines before or during the first few days after exposure (90). The non-governmental organization FIND recognizes 10 CE-marked rapid tests ready for use in the field (93, 94). Another report by John Hopkins Center for Health and Security lists a few RDTs currently approved for diagnostic use worldwide, but the antibody-RDT lateral flow assay by Cellex Inc. is the only RDT approved in the United States. This test was developed in the USA and China, with a sensitivity and specificity equal to 93.8 and 95.6%, respectively (90). The statistical significance was determined from studies in two Chinese hospitals on 128 SARS-CoV-2- positive patients and 250 SARS-CoV-2-negative patients (90). The test is currently available for purchase with a disclaimer from the FDA that the test alone cannot be used for definitive diagnosis.

In regards to the April 8 2020 WHO statement concerning immunoassays, the antibody-/antigen-detecting RDTs are only to be used for research purposes (95). According to recent data, these diagnostic tests do not produce results with appropriate reliability. Bruning et al. (96) claims that immunodiagnostic testing for influenza infections in cases with a viral load comparable to SARS-CoV-2 showed test sensitivity that varied between 34 and 80% (96). Also, due to their abrupt development, the majority of available rapid tests appear not to have been properly validated by relevant institutions and may pose a serious risk to diagnostic efficacy. The simplicity of the RDT means it is neither capable of quantifying the number of antibodies and therefore the phase of infection, nor is it able to ascertain whether these antibodies are part of long-term immunity to the virus. As of August 2020, it is unknown whether exposure imparts immunity after recovery. A 10 day or more delay in diagnosis is a serious diagnostic limitation and may not be of increased benefit to acute-state patients.

Another type of serologic testing for SARS-CoV-2 is a neutralization assay. It is a valuable method that detects antibodies capable of clearing the infection. The procedure consists of the infection of a special type of cell (e.g., VeroE6) with SARS-CoV-2, followed by incubation for 3–5 days at variable concentrations. During this time cells are titrated with human serum antibodies in order to detect the quantity of antibodies necessary to stop viral replication (90). A study performed by Zhou et al. demonstrated that 1:40–1:80 dilutions of IgG-positive sample were capable of neutralizing 100TCID_50_ (50% tissue-culture-infective dose) in SARS-CoV-2 cultures (21). Other than this result, it remains a relatively novel area of research, and information regarding its usefulness in the diagnosis of COVID-19 is sparse (90). Table 4 lists a number of exemplary testing kits currently available for diagnostic use.

**Table 4:** A list of exemplary diagnostic serologic assays currently used for SARS-CoV-2 (90).

**Type**	**Target**	**Author/company, country**	**Sensitivity**	**Specificity**	**Date of release**	**Phase of development**
Rapid diagnostic test	Solid phase immuno-chromatographic assay for IgG/IgM from blood or plasma	Aytu Biosciences/Orient Gene Biotech USA/China	87.9% IgG 97.2% IgM	100%	10-Mar	CE approved, awaiting FDA approval
	Lateral flow assay detecting IgG and IgM to the nucleocapside protein	Cellex Inc. USA	93.8%	95.6%	01-Apr	FDA approved
	Human venous whole blood, plasma from anticoagulated blood, or serum to detect IgG and IgM antibodies	Healgen Scientific LLC USA/China	96.7% IgG 86.7% IgM	98% IgG 99% IgM	01-Jun	FDA approved
	The target antigen is recombinant spike protein receptor binding domain	Hangzhou Biotest Biotech Co., Ltd China	91.6% IgG 92.5% IgM	99.5% IgG 98.1% IgM	04-Jun	FDA approved
Neutralization assay	A use of pseudovirus expressing the RBD of the spike protein to assay the ability of antibodies to block virus interaction with ACE2 receptors.	Genscript USA	93.0%	100	26-May	Research use only, CE approval
	SARS-CoV-2 and SARS-CoV-1 infection rate quantified using firefly luciferase reporter assay	Suhandynata et al. USA	96.6%	98.8	11-Jul	Pre-clinical
ELISA	Detects IgG specific to recombinant spike protein subunits 1 and 2 (S1 and S2)	DiaSorin Inc. USA	90.0–97.0%	98.0%	24-Apr	FDA approved
	Target antigen is recombinant nucleocapsid protein	Bio-Rad USA	98.0%	99.0%	29-Apr	EUA
	Target protein is a viral S1 region of the spike protein	Euroimmun AG Germany	0–10 days 13.9% 11–20 days 61.1% >21d 100%	100%	04-May	EUA
	Target antigen not stated	InBios International, Inc. USA	92.5%	98.5%	30-Jun	EUA

^*^EUA, Emergency Use Authorization.

#### Enzyme-Based Immunoassays

Enzyme-linked immunosorbent assay (ELISA) is currently one of the most popular commercially used enzyme based immunoassays. As indicated by the name, it is an assay using an enzymatic reaction to indicate a positive diagnostic result. Plates covered with viral antigens, prepared by the manufacturer, are incubated with the patient's serum. In cases where the specific IgG and IgM antibodies are present, the antibody-antigen binding complex will be visualized through an enzymatic reaction (colorimetric change, fluorescence, etc.) The whole procedure may take up to 5 h and requires a large sample of blood, plasma, or serum. It is both a qualitative and quantitative type of assay, thus has great potential for future diagnoses of SARS-CoV-2 infections. In a study of SARS-CoV-2-specific antibody responses in COVID-19 patients, Nisreen et al. used ELISA to demonstrate that severe viral infection resulted in higher antibody levels. Additionally, they claim that IgG seroconversion can be confirmed by applying the same technique in the second week of symptom onset (97). Another team proved that the accuracy of ELISA is dependent on the timing of the infection. On days 0–10 after symptom onset, ELISA showed <60% positive rate; the rate rapidly increased beyond that time frame for both IgM and IgG antibodies. Liu et al. evaluated immunoassays for detection of antibodies against SARS-CoV-2, and demonstrated that viral protein S (spike)-based IgM ELISA had statistically higher sensitivity than N (nucleocapsid)-based ELISA (*P* < 0.05) in detecting IgM antibodies. It is suspected that the level of immunogenicity of the S protein is the reason for the relatively higher specificity compared to the N protein (98).

Based on this data, the antigen-based ELISA will be a valuable diagnostic method for the fight against COVID-19. Currently, the NGO FIND lists 66 different immunoassay kits already commercialized, with 16 additional test kits in development (94). With supplemental research, these have the potential to become a powerful rapid diagnostic tool for the hospital setting. However, due to their complexity they, unlike RDTs, may not be used in the field. To reiterate, there is an insufficient amount of evidence-based research regarding ELISA's specificity and sensitivity for use in diagnostic confirmation, to be comparable with rRT-PCR assays.

A lesser known enzyme-based immunoassay that is nevertheless noteworthy is the chemiluminescent immunoassay (CLIA). Very similar to ELISA, it uses enzyme-labeled antibodies which activate an enzymatic reaction upon contact with their target. The photon of light emitted—luminescence—can then be quantified and directly corresponded to the volume of reagents. The benefit of this technique is its high sensitivity and the possibility of enhancing the reaction to allow for a larger threshold in samples with higher substrate concentration. CLIA can be used to detect versatile targets including IgM, IgG, and IgA, and there seems to have been a recent increase in trust for this technique among clinicians. A systematic review by Bastos et al. compared LFIA, ELISA, and CLIA tests, and the results suggest that CLIA exhibits the highest sensitivity and specificity for IgM and IgG in patients with COVID-19 (99). However, Bastos and colleagues also demonstrated that due to excessive discrepancies in test rests, none of the three techniques tested were reliable enough to be recommended for large-scale diagnostic purposes.

## Treatment Protocols

### Antiviral Approach

#### Lopinavir/Ritonavir + Ribavirin

After the 2002–2003 SARS outbreak, Chandwani and Shuter conducted an *in vitro* study using an engineered prototype of SARS-CoV to test lopinavir/ritonavir, protease inhibitors indicated for dual-therapy prophylaxis and treatment of HIV-1 (100). Lopinavir has higher potency but is less bioavailable, thus co-administration with ritonavir, which additionally inhibits cytochrome P450 34A, leads to prolongation of its action (101). They showed that this dual therapy inhibited viral cytopathic activity. Furthermore, Chu et al. conducted a non-randomized trial in 2004, placing two groups of patients on different regimens: the first group received ribavirin, a nucleoside inhibitor, in dual therapy with a reducing dose of corticosteroids; and the second group received lopinavir/ritonavir/ribavirin. It was shown that treatment group two had a decrease in viral load, fewer nosocomial infections and an increase in circulating lymphocytes, indicating a favorable outcome (102).

After the emergence of SARS-CoV-2, a randomized trial was conducted involving 199 severe cases. Ninety nine of these patients received lopinavir/ritonavir while 100 received standard supportive care. Detectable viral load in both groups was the same, and time to improvement after lopinavir/ritonavir was only decreased by 1 day, as compared to the group receiving standard care. The authors concluded that the treatment benefits of lopinavir/ritonavir were not established for severe illness (103). Similarly, an open-label randomized control trial by Cao et al. (ChiCTR2000029308), involving severe SARS-CoV-2 cases, compared lopinavir/ritonavir treatment with standard care alone, and they showed that the antivirals yielded no clinical benefits. Further trials are thus recommended to establish any possible benefits for patients suffering from less severe illness (104).

#### Comparative Study Between Favipiravir and Lopinavir/Ritonavir

Favipiravir, another RdRp inhibitor, is a broad antiviral with a mechanism of action that is hypothesized to either incorporate within viral RNA leading to chain termination, or bind to a conserved region of the RdRp and prevent nucleotide addition (105). Interferon-alpha (IFN-α) is an antiviral that binds to interferon receptors and activates signal modulators (JAK1/2). The phosphorylated interferon receptor binds to the signal modulators, resulting in immune modulation and antiviral protein transcription. In an open-label control study conducted by Cai et al., the antiviral activity of favipiravir + IFN-α was compared to that of lopinavir/ritonavir + IFN-α in patients with confirmed SARS-CoV-2 infection. They demonstrated that patients receiving favipiravir + IFN-α exhibited faster viral clearance and radiological improvement, compared to the other study group. Although yielding positive results, this was not a double-blind placebo-controlled randomized study, and more trials must be implemented before definitive conclusions can be drawn (106).

#### Comparative Study Between [Umifenovir + Lopinavir/Ritonavir] and Lopinavir/Ritonavir Alone

Umifenovir is a broad spectrum antiviral possessing dual properties: direct antiviral and virucidal action, as well as demonstrating virustatic effect through impedance of various stages of the viral life cycle (107). Umifenovir was shown by Stockman *et al*. to possess antiviral efficacy against SARS-CoV (108). Disease severity is known to be mostly related to a hyperactive immune response (109). Deng et al. conducted a study on a cohort of 33 patients with similar baseline characteristics, in which 16 patients received umifenovir + lopinavir/ritonavir, and 17 patients received only lopinavir/ritonavir. After 7 days of treatment, 75% of the first group tested negative for the virus whereas only 35% of the second group tested negative. Radiological investigation further supported the efficacy of the first group's treatment protocol. Deng and colleagues posit that a faster reduction in viral load prevents overstimulation of the immune system, thus diminishing the severity, duration and infectiveness of the illness (110). Zhu et al. compared the effectiveness and safety of umifenovir vs. lopinavir/ritonavir in 50 patients, of which 16 received umifenovir and 34 received lopinavir/ritonavir. After 14 days of treatment, no viral load was detected in the umifenovir group whereas 44.1% in the lopinavir/ritonavir group still had detectable levels. Clinical benefit was more apparent in the umifenovir group, and no side effects were observed in either group (111). An open-label randomized control trial (ChiCTR2000030254) was conducted by Chen et al. in which adult SARS-CoV-2 patients were administered either favipiravir or umifenovir. They showed that favipiravir significantly improved symptoms associated with cough and pyrexia. However, no clinical benefit could be observed when comparing viral clearance between the two therapies (112).

#### Multi-Antiviral Drug Testing

After isolating the genomic sequence of SARS-CoV-2, in particular that pertaining to RNA dependent RNA-polymerase (RdRp), Elfiky showed that the RdRp of SARS-CoV-2 exhibits 90.18% similarity with that of SARS-CoV. An RdRp model was engineered from the NCBI nucleotide protein database using the SARS-CoV RdRp genome as a template to test several antiviral drugs. To ensure high reliability of the model, a nucleotide comparison between the RdRp model and the SARS-CoV-2 RdRp was established, and was shown to yield 97.08% homology. The study sought to establish which of 24 compounds (Table 5) could bind to RdRp active sites and elicit inhibitory activity. Drug docking site analyses were interpreted using Protein-Ligand-Interaction-Profiler (PLIP). Five FDA-approved drugs showed very high affinity for RdRp, and could prove to be beneficial against SARS-CoV-2. Additionally, three drugs from the clinical trial by Elficky showed high affinity for RdRp, and these include IDX-184, setrobuvir and YAK. Drug side effects and toxicity are yet to be disclosed (117). Additionally, one antiviral drug that sparked interest was remdesivir. In a small cohort study, a 10 day course of remdesivir was administered to 53 patients with severe infection, and clinical improvement was observed in 68%. Fewer clinical benefits were observed in patients who received invasive ventilator support, and 13% of the patients, especially those who received invasive ventilation, died after the treatment course. Multiple factors impede the accurate measurement of remdesivir efficacy and these include preexisting conditions and duration of intubation. As a result, any clinical benefits need to be further investigated in future placebo-controlled trials (118). In a double blind placebo-controlled randomized trial (NTC04280705) Biegel et al. administered remdesivir to hospitalized SARS-CoV-2 patients presenting with lower respiratory tract involvement to assess its efficacy and safety. Remdesivir was shown to be superior to placebo in decreasing recovery time (119). However, a multicenter, randomized, placebo-controlled trial conducted by Wang et al. to assess the efficacy of remdesivir in patients with severe SARS-CoV-2 (NTC04257656) showed that remdesivir was of no clinical benefit compared to placebo (120). Lastly, a multicenter analysis involving a double-blind placebo-controlled trial (NTC04280705) was implemented to assess the efficacy and safety of remdesivir in the treatment of hospitalized SARS-CoV-2 patients. Preliminary results from this trial showed that patients receiving remdesivir had a faster time to recovery and a lower mortality rate when compared to the placebo group, and it is on the basis of this that the FDA issued authorization of remdesivir for emergency use on May 1, 2020.

**Table 5:** Drugs employed in multi-antiviral testing conducted by Elfiky, 2020.

**Native physiological compounds with high binding affinity for RdRp activity** GTP, UTP, CTP, and ATP		
**FDA-approved drugs**		**Mechanism of action**
Galidesivir	Nucleoside analog that binds RdRp	Causes structural changes that lead to termination of RNA elongation (113)
Remdesivir	Nucleoside analog	Is incorporated into viral RNA once metabolized, and prevents further addition of nucleotides (114)
Tenofovir	Nucleotide analog, reverse transcriptase inhibitor for HIV	Also inhibits HBV polymerase through competitive binding with deoxyribonucleotide substrate, causing termination
Sofosbuvir	Nucleotide analog inhibitor	Binds HCV NSP 5B on the RdRp; is incorporates into viral RNA once metabolized and causes chain termination (115)
Ribavirin		Activated by adenosine kinase to ribavirin-triphosphate-RTP; binds to the nucleotide binding site and prevents further nucleotide addition. Another mechanism of action is the inhibition of 2-O'-methyltransferase, resulting in disruption of the 5'CAP addition to viral mRNA (116)
**Clinical trials—mechanism of action unknown**.		
Uprifosbuvir, Setrobuvir, Balaprevir, 2'-C-methylcytidine, Valopectibine BMS-986094, MK0608, R7128, R1479, IDX-184, YAK, PSI-6130 and PSI-6206		

### Immune-Mediated Treatments

#### Administration/Induction of Interferons

Interferons (IFNs) are important cytokines with critical antiviral activity. Infected innate immune cells produce IFN which enable the JAK-STAT pathway, leading to recruitment of more NK cells and macrophages. As with SARS-CoV and MERS-CoV, NSP 1 of SARS-CoV-2 exhibits anti-IFN activity by targeting proteins of the JAK-STAT signaling pathway (121). IFN inhibition has been correlated to disease severity (122). Furthermore, IFN treatment has already proved effective against ss-RNA viruses, and is widely used in the treatment of HCV and HBV. Zhang et al. combined IFN-α and IFN-γ in a study conducted *in vivo* and *in vitro*. This combination demonstrated synergy, and smaller dosages were required than in IFN monotherapy. This suggests that reduction in unwanted side effects may be possible with the use of dual therapy (123). It was shown that SARS-CoV-2 evades detection from cytosolic RIG-I and MDA5, preventing the activation of IFN-I and the subsequent stimulation of innate cells. It is at this point that the importance of toll-like receptors (TLRs) should be emphasized. Negishi et al. demonstrated that viruses that evade cytosolic safeguards can be inhibited by TLR-3 action. TLR-3 functions to activate IFN-II, which in turn elicits an antiviral response (124). The use of TLR-3 agonists in mice by Shahabi Nezhad et al. showed promising results; they were able to increase levels of IFN-α/β/γ, which compensates for the inhibitory actions of SARS-CoV on signaling pathways (105) but further research is needed to determine if this may result in toxic overstimulation of the immune system (125). In an open-label randomized phase II trial by Hung et al., a triple combination of IFN-β-1b, lopinavir/ritonavir and ribavirin was administered to COVID-19 patients with mild to moderate disease. This combination proved to be safe and superior to lopinavir/ritonavir + ribavirin, with patients showing alleviation of symptoms, shortened duration of viral shedding, and shortened hospital stay. These promising results mean that future trials utilizing IFN-β-1b are warranted (126).

#### Use of Monoclonal Antibodies and Other Immune Modulators

##### Anti-C5a-antibody, eculizumab, and bevacizumab

The discovery of the SARS-CoV-2 specificity to MASP-2 of the MBL pathway opened the potential for prophylactic treatment against cytokine-mediated lung damage. It was previously shown in the literature that acute lung injury due to viral infection could be prevented by the use of anti-C5a-antibody treatment (127), and on the basis of this, Gao et al. used a recombinant C5a-antibody in an open-label trial involving severely ill patients The outcome of the first two recipients of the monoclonal antibody was described. Both patients showed improved oxygen saturation, increased lymphocyte count, decrease in inflammatory proteins, improvement in liver function, and alleviation of pneumonia. Although the use of anti-C5a antibody shows great promise, the trial is still ongoing and the final efficacy is yet to be disclosed (85). Another trial (NTC0428713) is currently assessing the potential of eculizumab to reduce mortality in 19 patients. In a similar mechanism of anti-inflammatory action, eculizumab inhibits C5 cleavage thus preventing the release of C5a. For the treatment of ARDS, a clinical trial (NCT04275414) is comparing the therapeutic potential and side effects of the monoclonal antibody bevacizumab for critical COVID-19 patients. By targeting vascular endothelial growth factor (VEGF), an angiogenic factor, bevacizumab may prevent the disruption of the vascular barrier that causes edema and lung injury (128).

##### 47D11

A preliminary study conducted by Wang et al. (129) identified potential antibodies with the capacity to neutralize SARS-CoV-2. The S protein ectodomains of both SARS-CoV and SARS-CoV-2 were expressed in HEK-293T cells using plasmid transduction. Similarly, HEK-293T cells were transfused with plasmids containing SARS-CoV1/2 in S protein subdomains tagged with either the mice or human Fc portion of IgG. H2L2 mice antibodies were produced through gradual immunization with the SARS-CoV S protein ectodomain. The spleen and lymph nodes were then harvested to produce hybridomas. Of the 51 samples, only one of the hybridomas (47D11) exhibited cross-reactivity SARS-CoV and SARS-CoV-2 S proteins. The chimeric antibody was reformatted and expressed as a fully human IgG. It was shown that this novel IgG tightly binds the conserved RBD of the SARS-CoV-2 S protein in infected cells and neutralized the virus in VeroE6 cells. This represents the very first human monoclonal antibody that is able to fully neutralize SARS-CoV-2 (129). This cross-neutralizing antibody, due to a conserved epitope region on the spike protein, could be the key to preventing future *betacoronavirus* outbreaks. 47D11 has recently completed phase I clinical trials (NCT04411628) to establish dosing in hospitalized patients with SARS-CoV-2 under long-term follow up. Now undergoing phase II trials (NCT04427501), the monoclonal antibody will be tested on ambulatory patients, with results estimated to be available in September.

##### Hrs-ACE-2

As stated earlier, the ACE-2 receptor is crucial for viral attachment and entry. An *in vitro* experiment conducted by Monteil et al. exposed VeroE6 cells to varying concentrations of plaque forming units (PFU) from SARS-CoV-2 in the presence and absence of human recombinant soluble ACE-2 (hrsACE-2). The infection was inhibited 15 h after introduction of the virus. The experiment was repeated using human capillary organoids and human kidney organoids, and the same inhibitory actions of hrsACE-2 were observed. It is important to note the dose-dependent nature of this inhibitory action. Hrs-ACE-2 has already undergone phase I and II clinical trials for the treatment of ARDS (130), and Monteil et al.' findings suggest that hrs-ACE-2 could be a potential therapeutic agent against SARS-CoV-2 and phase II trials (NCT04335136) are currently underway in various European countries (131).

##### Glucocorticoids

In an open label trial by the RECOVERY Collaborative Group, 2,014 hospitalized patients received either low dose dexamethasone or standard care alone by random assignment. It was observed that the group of patients on mechanical ventilation who received dexamethasone exhibited lower mortality rates compared to those receiving standard care alone (132). While other studies further support the role of glucocorticoids in the reduction of mortality (133) some reported conflicting results and showed no clinical benefit or even harm to the patient (134). A systematic review of 15 studies concluded that while critically ill patients are more likely to benefit from glucocorticoid therapy, their use was associated with increased mortality as it resulted in longer hospital stays and increased tendency toward serious nosocomial infections (135). Clinical trials are currently ongoing to assess the risk vs. benefit for the use of glucocorticoids in the treatment of COVID-19.

##### Tocilizumab

Cytokine storm remains the main cause of acute lung injury and organ damage in the course of SARS-CoV-2 infection. It is chiefly caused by GM-CSF and IL-6. IL-6 receptors exist in two forms: the soluble form (sIL-6) and the membrane bound (mIL-6). To initiate pro-inflammatory action, IL-6 binds to sIL-6 and the complex binds to gp130 to complete signal transduction. Due to this, the therapeutic use of the IL-6 antagonist tocilizumab, which binds to both sIL-6 and mIL-6, was suggested. Xu et al. qualified 21 critical patients in a trial with tocilizumab. Clinical symptoms, radiological findings and laboratory values all improved after treatment, and 19 patients were successfully discharged (136). In an open label study by Morena et al., however, 51 critically ill SARS-CoV-2 patients were treated with tocilizumab. All patients presented with decreased oxygen saturation and an increase in plasma IL-6. While positive results were observed with tocilizumab rapidly decreasing inflammatory markers, no clinical benefit was reported as patients quickly developed life-threatening bacterial and fungal infections (137). Jordan et al. conducted another study administering 27 critical patients with a single dose of tocilizumab. This resulted in significant decrease in inflammatory proteins and reduction in fever. Twenty two patients receiving mechanical ventilation were able to be extubated and vasopressors discontinued. Two patients died, and the authors report that these patients were already in severe septic shock due to SARS-CoV-2-related pneumonia, and were unresponsive to vasopressors. Four patients did not respond to the medication and had poorer outcomes. While the results are promising and in line with the findings by Xu et al., limitations of this work include the lower-than-recommended dose of tocilizumab, chosen by the authors due to drug shortage, and the absence of a control group (138). A placebo-controlled randomized clinical trial is still necessary before recommendations can be made.

##### IL-1 inhibitors and Anakinra

Historically, IL-1 has been shown to act as a pro-inflammatory cytokine with actions on several immune cells (139, 140). IL-1 inhibitors competitively bind IL-1 receptors in various tissues to inhibit the inflammatory cascade. A phase III trial by Shakoory et al. assessed the efficacy of IL-1 inhibitors on septic patients presenting with hepatobiliary dysfunction and or/disseminated intravascular coagulation. Promisingly, IL-1 blockade was associated with decreased mortality (141). More recently, a study by Monteagudo et al. showed that IL-1 inhibitors were also effective in calming cytokine storm in hemophagocytic lymphocytosis patients (142). Huet et al. also published a cohort study of 52 critically ill SARS-CoV-2 patients given Anakinra (an IL-1-inhibitor) and supportive care, while a control group of 44 patients received only supportive care. The outcome of the study was positive: no serious side effects were observed and Anakinra use reduced the need for mechanical ventilation. Overall mortality was reduced (143). A similar study was conducted by Cavalli et al. using IL-1 inhibitors, with the same positive outcome (144).

##### Convalescent plasma

Convalescent plasma has been employed and has shown promise in the treatment of SARS, MERS and Influenza (145–147). In a study by Duan et al., 10 severely ill patients were transfused with 200 ml of convalescent plasma harvested from donors who have recovered from SARS-CoV-2 and had antibody titers above 1:640. Within 1 to 3 days, symptoms had disappeared in all 10 patients, and radiological investigation showed improvement after seven days. Viral load was undetectable by RT-PCR in 7 patients and no adverse side effects were detected (148). Shen et al. treated five COVID-19 patients with convalescent plasma. All five patients were critically ill and intubated, presenting with ARDS and pneumonia and experiencing high viral load despite treatment with antivirals. The outcome was largely positive: ARDS was resolved in four patients within 12 days, and three patients were able to be weaned off mechanical ventilation within 2 weeks. Three patients were discharged while two remained stable in recovery (149). Joyner et al. recently made an assessment of the safety of convalescent plasma in 20,000 COVID-19 patients. The incidence of serious adverse events including transfusion reactions, cardiac events and thrombotic events was low. Mortality rates were shown to be higher in critical patients receiving mechanical ventilation or those in septic shock. The conclusion drawn from the study provided evidence that the administration of convalescent plasma in a hospital setting was safe and that early administration is more likely to reduce fatality rates (150).

##### IL-38 and IL-37

It has been previously shown that SARS-CoV induces viroporin production in the host cell membrane to facilitate virion release. Viroporin 3a has been associated with NLRP3 (Nod-Like R family, pyrin domain 3) inflammasome activation (151), which induces the production of pro-inflammatory cytokines such as IL-1-β and IL-18 via the Gasdermin D (GSMD) pathway (151, 152). Like SARS-CoV, SARS-CoV-2 induces the production of large amounts of IL-1-β (33). On the basis of this, a possible treatment could be IL-38, a member of the IL-1 family. When placed with activated peripheral mononuclear cells, IL-38 demonstrates suppressive and anti-inflammatory effects through the inhibition of IL-1, IL-6, and TNF production (153). Another member of the IL-1 family is IL-37. Both *in vitro* and *in vivo* studies showed that IL-37 acts as a negative regulator of inflammation, aiding in the protective actions exhibited by TGF-β on dendritic cells and thus attenuating the T cell response (154). Both IL-38 and IL-37 could potentially be valuable in the treatment of COVID-19 (155).

##### Baricitinib

Baricitinib is a janus kinase (JAK) 1/2 inhibitor used to treat rheumatoid arthritis. JAK 1/2 inhibition prevents activation of pro-inflammatory cytokines such as GM-CSF, IL-2, IL-6, IL-12, and IL-23. Adaptor-related protein complex 2 (AP2)-associated protein kinase I (AAK1) induces receptor-mediated endocytosis. Baricitinib has been shown to have very high affinity for AAK1, thus could feasibly inhibit both cytokine storm and viral entry to the cell (156). In a small cohort study, Titanji et al. administered baricitinib and hydroxychloroquine to 15 patients with moderate to severe COVID-19. Twelve of the 15 patients recovered, and vitals and inflammatory markers were seen to improve after baricitinib was initiated. Two patients however developed serious bacterial or fungal infections due to prolonged ICU stay (157). A phase III double blind placebo-controlled randomized trial involving baricitinib (NCT04421027) is currently ongoing to assess the efficacy and safety of the drug as a potential immune inhibitor preventing cytokine storm and viral entry.

SARS-CoV-2 is the most structurally and genetically similar to SARS-CoV, thus findings from monoclonal studies on SARS-CoV have been utilized to target the shared aspects between the strains. The monoclonal antibodies shown in Table 6 are engineered to specifically bind to different domains on S1 or S2. Though none have progressed to clinical trials, they show promise *in vitro* and *in vivo* against SARS-CoV and SARS-CoV-2 (164).

**Table 6:** Previously discovered monoclonal antibodies for SARS-CoV.

**Monoclonal antibody**	**Mechanism of action**
80R	Binds S1 and prevents interaction with ACE-2 (158)
CR3014 CR3022	Synergistic action; bind S1 and prevent interaction with ACE-2; prevent immune escape (159, 160)
F26G18 F26G19 M396	Bind linear epitope of S1; Bind conformational epitope of S1; Inhibit interaction of S1 with ACE-2 (161)
1A9	Binds HDR domain of S2 and prevents interaction of receptor *in vitro* (162)
201	Binds S1 and prevents interaction with ACE-2 (163)
**Monoclonal antibodies studied for the treatment of SARS-CoV-2**	**Function**
LY-CoV555	IgG that binds to a conserved region in the RBD of the S protein and fully neutralizes SARS-CoV and SARS-CoV-2 (129) [NCT04411628] [NCT04427501]
Anti-C5a antibody	Prevents cytokine-mediated lung damage (85, 127)
Hrs-ACE-2 (APN01)	ACE-2-receptor decoy preventing infection (130, 131) [NCT04335136]
Tocilizumab	IL-6 antagonist that binds both sIL-6 and mIL-6 thus preventing cytokine storm (136, 138)
Convalescent plasma	Preformed IgG harvested from cured patients (148, 149)
Bevacizumab	Targets VEGF, preventing vascular barrier permeability thus inhibiting the development of ARDS [NCT04275414] (128)
Anakinra	IL-1-inhibitor used to reduce effects of the cytokine storm in SARS-CoV-2 (143)

##### Mesenchymal stem cells

The immunomodulatory potential of mesenchymal stem cells (MSC) was first identified by Luk et al. in 2016, and then by Gonçalves et al. in 2017. MSCs can be isolated from peripheral blood, bone marrow, the umbilical cord or the placenta, and after expansion, administered to patients with cytokine storm and sepsis. (165, 166). The potential for MSCs to confer clinical improvement to COVID-19 patients was investigated by Leng et al., who administered MSCs to seven patients while three received a placebo. Symptoms associated with infection and impaired pulmonary function improved 2 days after MSC infusion, and full recovery was achieved 10 days post-infusion. The authors observed a significant decrease in inflammatory proteins in the MSC group on the third day post-infusion, while the control group did not improve. It should be noted that the expanded MSCs did not express ACE-2 or TMPRSS2, further indicating MSC therapy as a viable option for future investigation (167).

## Adjuvant Therapy

### Anticoagulant Therapy

Thromboembolism as a result of endothelial injury in the course of infection is a serious and fatal complication in critically ill patients (168–170). Tang et al. enrolled 449 COVID-19 patients with severe disease for a study in which 99 patients who received low molecular weight heparin for a week or more. These patients were associated with better prognosis than the patients who were not administered anticoagulant therapy (171). Helms et al. followed 150 ICU patients in a multicenter prospective cohort study. Thromboembolic events were observed in 16.7% of patients despite prophylactic and therapeutic use of anticoagulants. The authors noted that pulmonary embolism was diagnosed a few days after ICU admission and was more common in ARDS patients. They conclude that although other papers have reported the effectiveness of heparin, a higher anticoagulant target should be implemented with other anticoagulants such as anti-Xa (172). The pathogenesis of thromboembolism in the course of COVID-19 is still unclear. One of the proposed pathways implicates ARDS: the profound hypoxemia and vasoconstriction may lead to vascular occlusion (173). Another proposed mechanism is that unlike in a healthy lung, a diseased lung is unable to maintain the balance between fibrinolysis and coagulation, thus resulting in decreased action of tissue plasminogen activators (tPA) (174). More randomized clinical trials are currently ongoing to assess the efficacy of various anticoagulants.

### Vitamin C Supplementation

In a 2014 pilot study by Fowler et al., critically ill septic patients were given either a vitamin C infusion or a placebo. A dramatic decline in inflammatory markers was observed in the vitamin C group, and their sequential organ failure assessment (SOFA) score was decreased compared to the placebo group. There were also no adverse events observed with the vitamin C group (175). In 2019, Fowler et al. published on the use of vitamin C vs. placebo in septic patients with ARDS, and they concluded that the vitamin C infusion did not improve either inflammatory markers or organ dysfunction score (176). Several clinical trials are ongoing verifying the benefits of vitamin C in the treatment of COVID-19.

## Unorthodox Methods of Treatment

### Hydroxychloroquine

Widely used as an antimalarial drug, hydroxychloroquine has been shown to possess broad antiviral action, including effectiveness against HIV-1 and Influenza type A and B. Its antiviral activity has been tested on SARS-CoV-2. In preventing the glycosylation of the ACE-2 receptor, hydroxychloroquine effectively prevents viral entry (177). Furthermore, it has been shown to alkalinize the organelle, which serves to prevent the formation of mature endosomes required to shield the virus from immune cells, and for replication (178). Apart from its broad antiviral activity, hydroxychloroquine has proven to be adequately anti-inflammatory, interfering with NLRP3 activation and impairing the production of pro-inflammatory cytokines, specifically IL-1-β (179). In an open-label non-randomized clinical trial by Gautret et al., SARS-CoV-2-positive patients were divided into three groups: group 1 receiving hydroxychloroquine, group 2 receiving hydroxychloroquine + azithromycin (antibiotics were given at the discretion of the physician to prevent opportunistic infections), and group 3 not receiving hydroxychloroquine. The primary outcome in group 1 and 2 was viral clearance within 3 to 6 days, but greater results were achieved when hydroxychloroquine was combined with azithromycin (180). Azithromycin, a bacteriostatic agent, has shown antiviral activity against Zika virus, Ebola virus (181) and RSV. Its antiviral mechanism of action, with regards to RSV, is hypothesized to be in decreasing the expression of fusion proteins in airway epithelium (182). In a study conducted by Million et al., patients suffering from early SARS-CoV-2 infection were treated with hydroxychloroquine and azithromycin; this combination proved to efficaciously reduce the viral load and was deemed safe with minimal risk of complications (183). Chen et al. also showed that hydrochloroquine was safe and efficacious in the treatment of mild disease (184). The efficacy of hydrochloroquine with or without azithromycin has since been disputed. In a recent open-label randomized control trial, the efficacy and safety of hydroxychloroquine + standard care vs. standard care alone was assessed by Wei et al. in patients with mild to moderate COVID-19. The group receiving hydroxychloroquine was shown to suffer more adverse effects, and there was no observed clinical benefit compared to patients given standard care alone (185). Molina et al. also concluded that while hydroxychloroquine proved to be beneficial in past studies, the combination of hydroxychloroquine and azithromycin for severe SARS-CoV-2 infections resulted in inadequate viral clearance, and the clinical benefits previously seen in patients with mild to moderate illness were not observed in hospitalized patients with severe disease (186).

### Ivermectin

This broad spectrum antiparasitic agent has been shown to possess antiviral activity. *In vitro* studies indicate that ivermectin prevents non-structural proteins of both dengue virus (187) and HIV-1 (188) from interacting with the importin α/β1 on the host cell, which prevents viral integration. It has been shown that the SARS-CoV nucleocapsid (N) protein integrates with the nucleus and nucleolus, and prevents cytokinesis of the host cell via importin-α/β1. The exact role of the N protein in the cell cycle is not known, but it is postulated that this structural protein enters the nucleolus to promote viral replication and encourage suitable conditions for viral packaging (189). An *in vitro* experiment by Caly et al. showed that a single dose of ivermectin administered to inoculated Vero cells effectively controlled viral replication within 1 to 2 days, which may prove beneficial for newly infected patients. It was hypothesized that like in other viruses, ivermectin's antiviral action against SARS-CoV-2 is derived from the inhibition of importin-α/β1. (190). Alam et al. treated 100 mild to moderately ill patients with a combination of ivermectin and doxycycline. Symptom improvement was observed after 72 h following treatment, and no side effects were noted. This study however makes no conclusion on the efficacy and safety of this therapy as it is not a place-controlled randomized clinical trial (191). Similarly, Caly et al.' results, while promising, cannot be applied until safety margins have been established in further clinical trials.

### Tecoplanin

This glycopeptide antibiotic used in the treatment of Gram-positive bacterial infections has been shown to possess antiviral activity against Ebola, MERS, SARS and HIV-1. It is believed that teicoplanin interferes with endosome formation through alkalization. Cleavage of the S protein by cathepsin in the late endosome is inhibited, which in turn prevents the release of viral RNA (192). Baron et al. showed that the cathepsin L sequence is conserved in SARS-CoV, suggesting that teicoplanin could be a key treatment in patients who are diagnosed early with SARS-CoV-2 (193).

## Future Treatments

There have been tremendous advancements in the field of immunotherapy since the development of chimeric antigen-receptor T cells (CAR-T). These cells differentiate from our basic T-cells by overcoming T-cell control safeguards and therefore express fewer exhaustion markers PD-1, TIM3, and Lag3. Furthermore, they are capable of differentiating into terminal effector T-cells responsible for pathogen and tumor destruction. CAR-T cell treatment has already shown great advances in oncology, inducing long-term remission in patients suffering from acute lymphoblastic leukemia (194). It is currently being investigated for the treatment of viral infections such as HIV-1, HBV, and HCV. Development of CAR-T cells specific to HIV-1 infection has now entered clinical trials (195).

CRISPR-Cas (Clustered Regularly Interspaced Short Palindromic Repeats), a mechanism evolved to protect against bacteriophages has shown great promise as a genetic editing tool. *In vitro* studies have used lentiviral vectors consisting of Cas9 (CRISPR-associated proteins) and sgRNA specific CCR5 (single guided RNA responsible for the detection of the genome of interest) against CD4+ T cells susceptible to HIV-1 infection. The viral vector was able to disrupt the CCR5 gene in the CD4+ cells thus inhibiting HIV-1 entry (196). In a novel study, the use of the CRISPR-Cas system against human lung epithelium infected with SARS-CoV-2 yielded positive results: CRISPR-Cas was able to be transfected in the lung epithelium to degrade the virus (197). This method was shown to possess protective actions against the known pathogenic coronaviruses. Gene editing and CAR-T cell therapy open a new frontier in future treatment modalities.

## Conclusion

The rapid spread of SARS-CoV-2 poses a threat of global proportions. Time is of the essence, and the discovery of accurate diagnostic methods and treatment protocols are imperative in preventing further spread of this pathogen. Similarities between SARS-CoV-2 and its predecessor have formed the framework upon which diagnostic and treatment approaches to the novel virus are based. RT-PCR primers, based on SARS-CoV and MERS-CoV, have proven to be highly sensitive and specific, though not without their flaws. Time consuming and prone to producing false negative results, this has led to the employment of more efficient testing methods such as serologic tests and enzyme-based assays, capable of quantifying infected patients on a large scale. To date, there are still no therapies specifically targeting SARS-CoV-2. While many FDA-approved antivirals on the market have had success in patients presenting with differing degrees of illness severity, the development of specific antivirals remains an area of active research. On the other hand, immunotherapy has been shown to be effective, particularly with the discovery of hrs-ACE-2 and other promising immune modulators, the development of the 47D11 monoclonal antibody capable of neutralizing SARS-CoV-2, as well as MSC therapy. The non-traditional use of anti-malarial agents had previously showed great promise but have now proven to lack adequate antiviral action and have been associated with severe complications. Until guidelines are updated following the multitude of ongoing clinical trials, standard care remains the main treatment modality. Rigorous research with regards to this pandemic not only adds to the scientific literature, but is critical for public health policy surrounding future outbreaks. Only with collaborative research efforts and dissemination of knowledge may we interrupt exponential transmission of disease and maintain human losses at the minimum.

## Author Contributions

KK and JC: conceptualization. KK, NP, and DH: investigation and resources. KK, NP, JC, and DH: writing—original draft preparation. KK, JC, and MK: writing—review and editing. JC and MK: visualization. MK and AM: supervision and project administration. All authors contributed to the article and approved the submitted version.

## Conflict of Interest

The authors declare that the research was conducted in the absence of any commercial or financial relationships that could be construed as a potential conflict of interest.
